# Supplementary material for: Cryo-EM structure of an amyloid fibril formed by full-length human SOD1 reveals its conformational conversion
Source: Nat Commun. 2022 Jun 17;13:3491. doi: 10.1038/s41467-022-31240-4 (PMC9205981; doi:10.1038/s41467-022-31240-4)
Supplement: Supplementary file 1 — Supplementary Information [file 41467_2022_31240_MOESM1_ESM.pdf]

Supplementary Figures

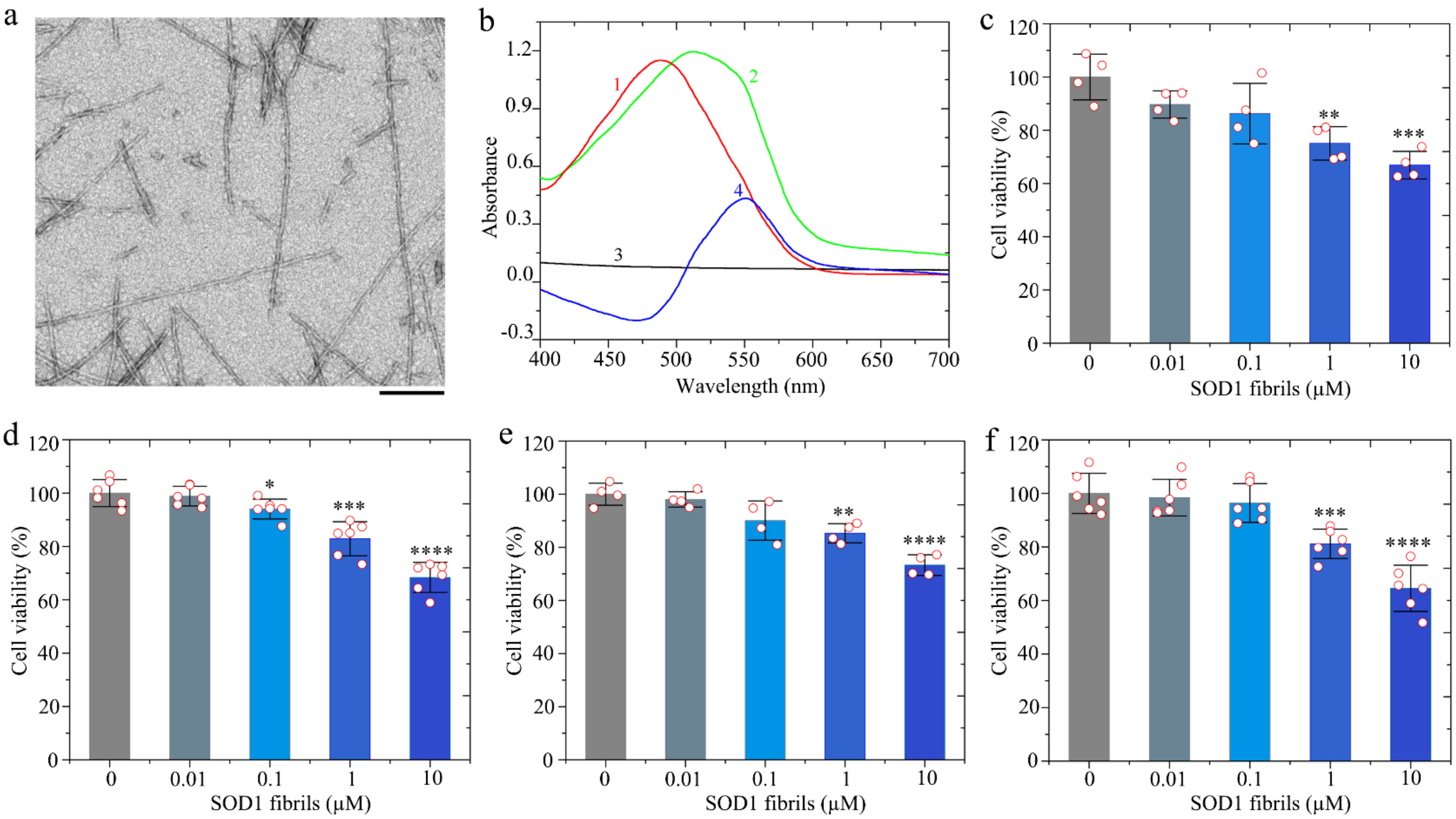

Supplementary Figure 1.

SOD1 forms cytotoxic amyloid fibrils under reducing conditions.

a A negative-staining TEM image of amyloid fibrils assembled from recombinant, full-length apo human SOD1 in 20 mM tris-HCl buffer (pH 7.4) containing 5 mM TCEP. Scale bar, 200 nm. **b** Amyloid fibrils from recombinant, full-length apo human SOD1 analyzed by Congo red binding assays. The absorbance spectrum 2 (SOD1 fibril + Congo red, green) minus the absorbance spectrum 1 (Congo red alone, red) with the maximum absorbance at 490 nm minus the absorbance spectrum 3 (SOD1 fibril alone, black) equals the difference spectrum 4 (blue) with the maximum absorbance at 550 nm. Congo red binding assays were carried out at 37°C. These experiments were repeated three times with different batches of fibrils and similar results. **c**, **d** Cytotoxicity of SOD1 amyloid fibril seeds to SH-SY5Y cells assessed by the MTT

assay (**c**) and the CCK8 assay (**d**). **e**, **f** Cytotoxicity of SOD1 amyloid fibril seeds to HEK-293T cells assessed by the MTT assay (**e**) and the CCK8 assay (**f**). Cells were treated with indicated concentrations of SOD1 fibril seeds for 2 days. The cell viability (%) (open red circles shown in scatter plots) is expressed as mean ± SD of the values obtained in $n = 4$ (**c**, **e**) or 6 (**d**, **f**) biologically independent experiments. 0.01 (blue grey), 0.1 (light blue), 1 (blue), and 10 (blue) μM SOD1 fibril seeds, (**c**) $p = 0.085$, 0.10, 0.0034, and 0.00058, respectively, (**d**) $p = 0.67$, 0.042, 0.00043, and 0.0000013, respectively, (**e**) $p = 0.46$, 0.057, 0.0017, and 0.000083, respectively, and (**f**) $p = 0.71$, 0.42, 0.00057, and 0.000018, respectively. SH-SY5Y or HEK-293T cells treated with 20 mM Tris-HCl buffer (pH 7.4) containing 5 mM TCEP for 2 days were used as a control (gray). p-values were determined using two-sided Student's t-test. Values of $p < 0.05$ indicate statistically significant differences. The following notation is used throughout: *$p < 0.05$; **$p < 0.01$; ***$p < 0.001$; and ****$p < 0.0001$ relative to the control. Source data are provided as a Source Data file.

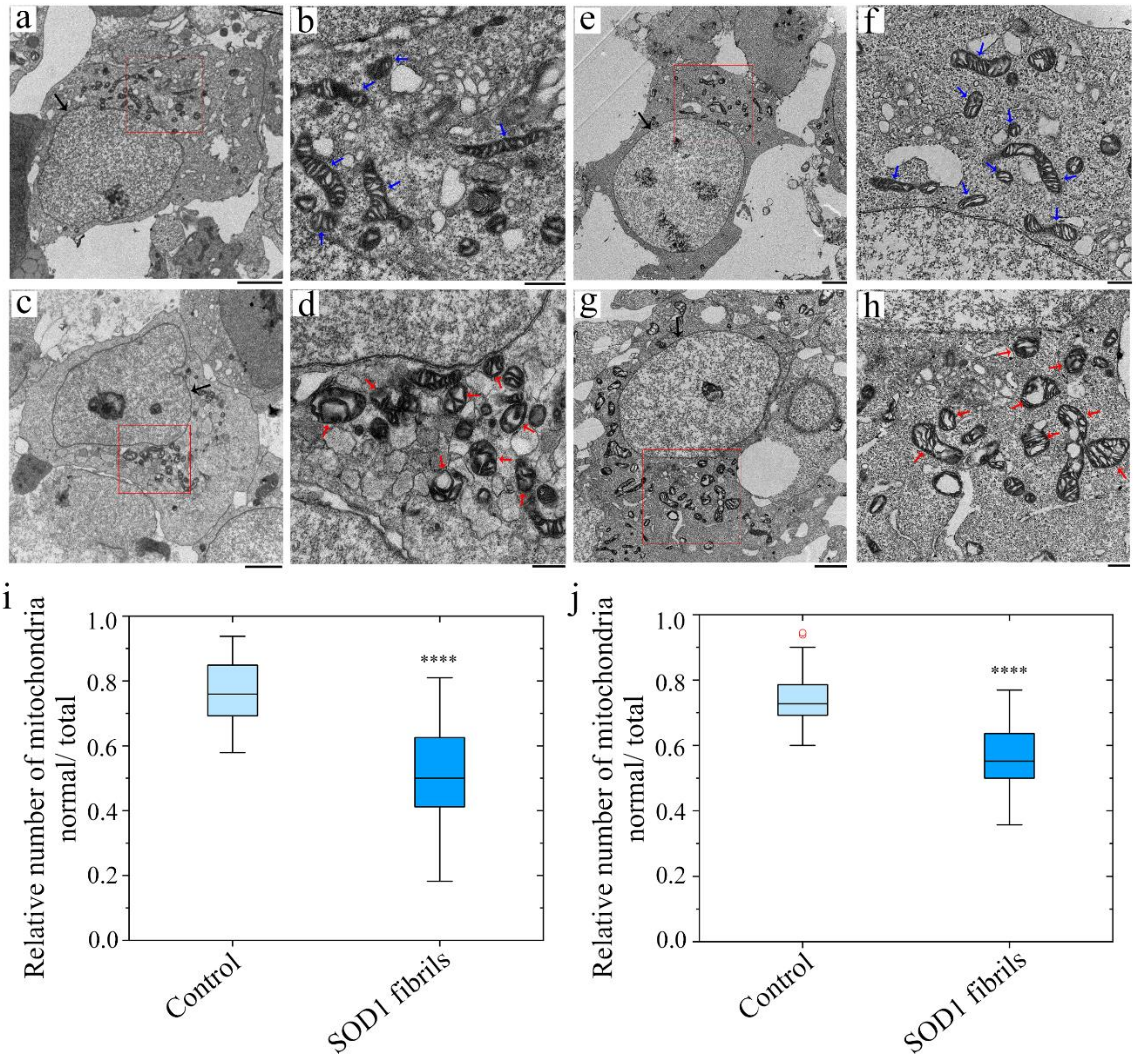

Supplementary Figure 2.

Treatment of cells with SOD1 amyloid fibril seeds causes severe mitochondrial impairment.

a–**h** SH-SY5Y (**a**–**d**) and HEK-293T (**e**–**h**) cells were cultured for 1 day and then incubated with 0 (**a**, **b**, **e**, and **f**) or 10 (**c**, **d**, **g**, and **h**) μM SOD1 fibril seeds for 3 days. The enlarged regions (**b**, **d**, **f**, and **h**) show 12-fold enlarged images from (**a**, **c**, **e**, and **g**), respectively, and display the detailed structures of mitochondria in cells. Nuclei are highlighted using black arrows (**a**, **c**, **e**, and **g**). The morphology of normal mitochondria in SH-SY5Y (**b**) and HEK-293T (**f**) cells incubated with 0 μM SOD1 fibril seeds, which are highlighted by blue arrows, was tubular or round. 10 μM SOD1 fibril seed treatment caused severe mitochondrial impairment in SH-SY5Y (**d**) and HEK-293T (**h**) cells.

Most of the mitochondria in the cells (~50%) became swollen and vacuolized, which is highlighted by red arrows. Samples were negatively stained using 2% uranyl acetate and lead citrate. The scale bars represent 2 μm (**a**, **c**, **e**, and **g**) and 500 nm (**b**, **d**, **f**, and **h**), respectively. **i**, **j** Box plots showing the quantification of TEM images in $n = 34$ SH-SY5Y (**i**) and 30 HEK-293T (**j**) cells examined over 3 independent experiments. The boxes (blue) extend from the 25th to 75th percentile (quantiles 1 and 3). Minima, maxima, centre, and bounds of box represent quantile 1 minus 1.5 × interquartile range, quantile 3 plus 1.5 × interquartile range, median (black line), and quantiles 1 and 3, respectively. Bounds of whiskers (minima and maxima) and outliers (open red circles). Cells treated with 20 mM Tris-HCl buffer (pH 7.4) containing 5 mM TCEP for 3 days were used as a control. A significantly lower number of normal mitochondria was observed in SOD1 fibril-treated cells than did in control cells treated by Tris-HCl buffer containing TCEP (**i**, $p = 0.000000000014$, and **j**, $p = 0.000000000048$). p-values were determined using two-sided Student's t-test. Values of $p < 0.05$ indicate statistically significant differences. The following notation is used throughout: $*p < 0.05$; $**p < 0.01$; $***p < 0.001$; and $****p < 0.0001$ relative to control. Source data are provided as a Source Data file.

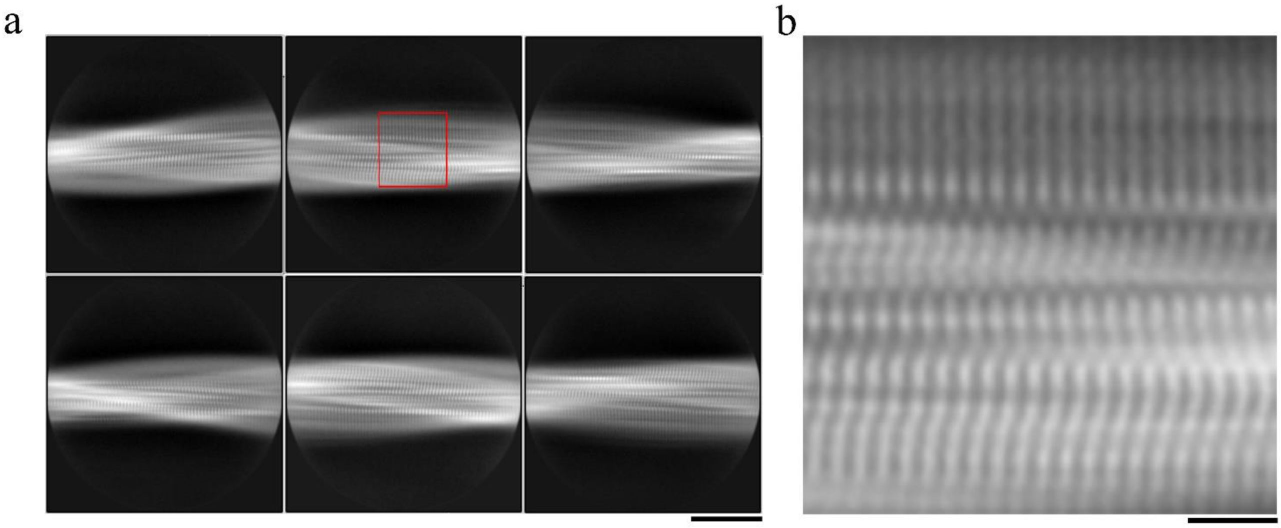

Supplementary Figure 3.

Cryo-EM images of SOD1 fibril.

a Reference-free 2D class averages of SOD1 fibril showing two protofilaments intertwined. Scale bar, 10 nm. **b** Enlarged image of (**a**) showing two protofilaments arranged in a staggered manner. Scale bar, 2 nm.

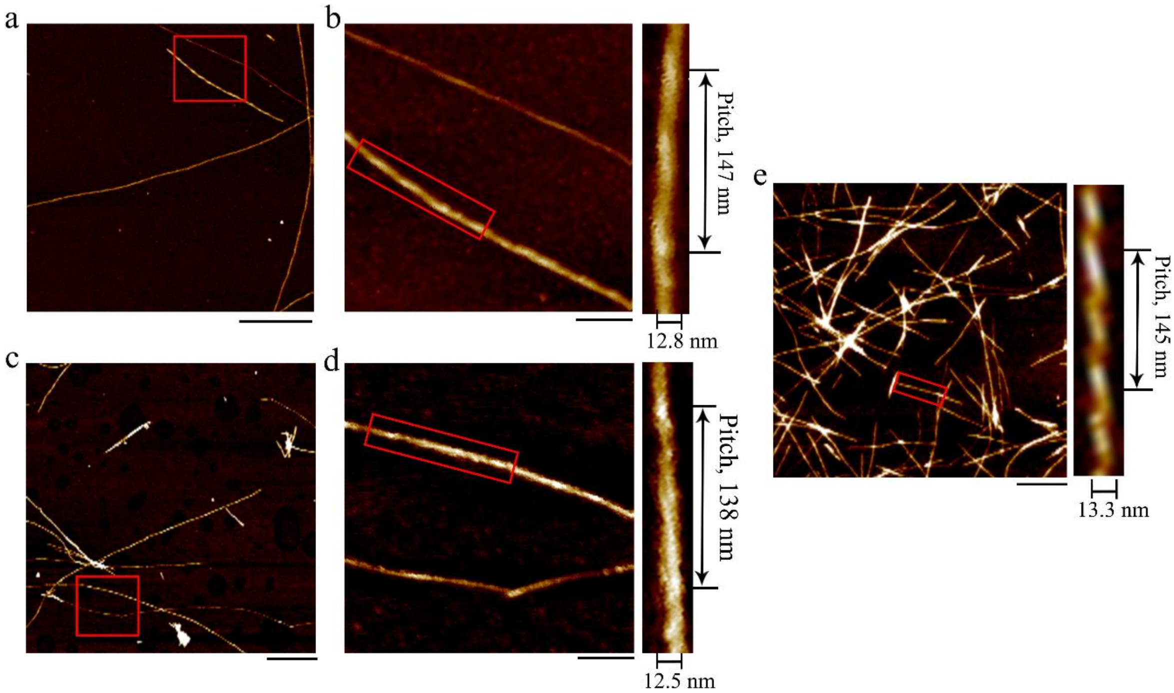

Supplementary Figure 4.

High-resolution AFM images of SOD1 fibrils.

a, **c**, and **e** AFM images of amyloid fibrils assembled from recombinant, full-length apo human SOD1 under reducing conditions. The enlarged regions (**b** and **d**) show 16-fold enlarged images from (**a** and **c**, red square), respectively, and visualize the detailed morphology of SOD1 fibrils. Enlarged sections of **b**, **d**, and **e** (right) showing the SOD1 fibril intertwined into a left-handed helix, with a fibril full width of 12.9 ± 1.0 nm and a helical pitch of 146 ± 5 nm. The scale bars represent 500 nm (**a**, **c**, and **e**) and 100 nm (**b** and **d**), respectively. The helical pitch and fibril width were measured and expressed as the mean ± SD of values obtained in n = 8 biologically independent measurements. Source data are provided as a Source Data file.

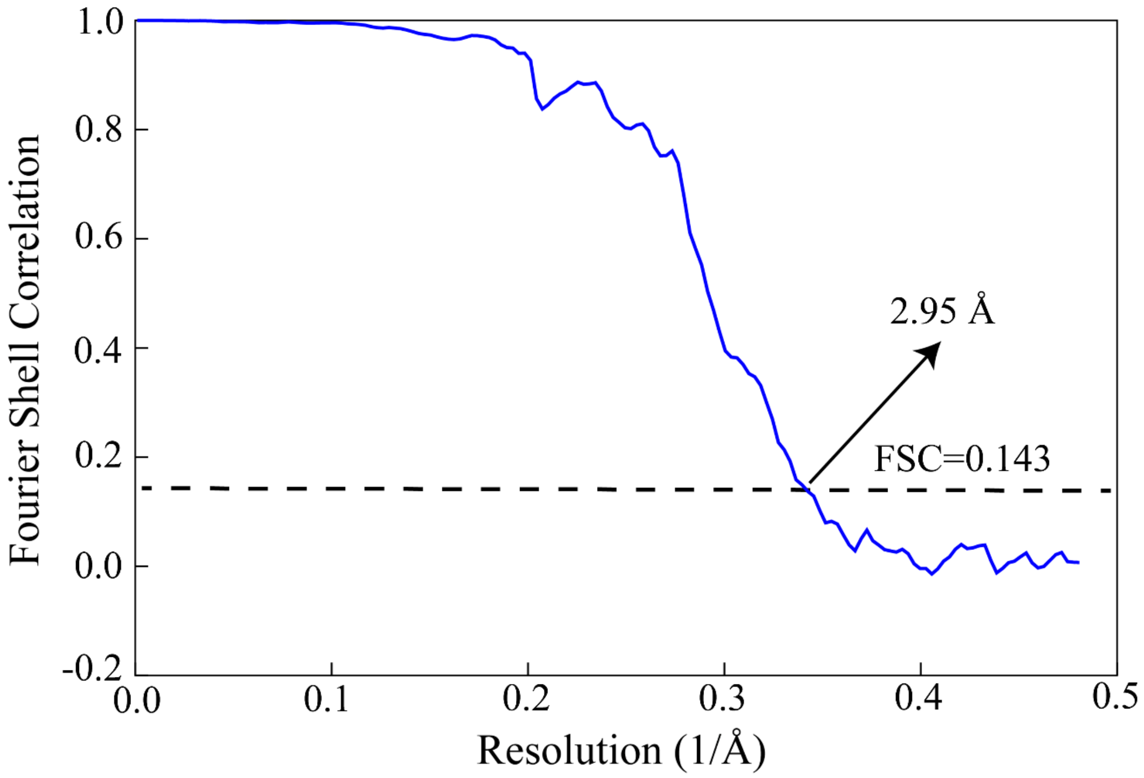

Supplementary Figure 5.

Global resolution estimate for the SOD1 fibril reconstructions.

The reconstruction was reworked and gold-standard refinement was used for estimation of the density map resolution. The global resolution of 2.95 Å was calculated using a Fourier shell correlation (FSC) curve (blue) cut-off at 0.143.

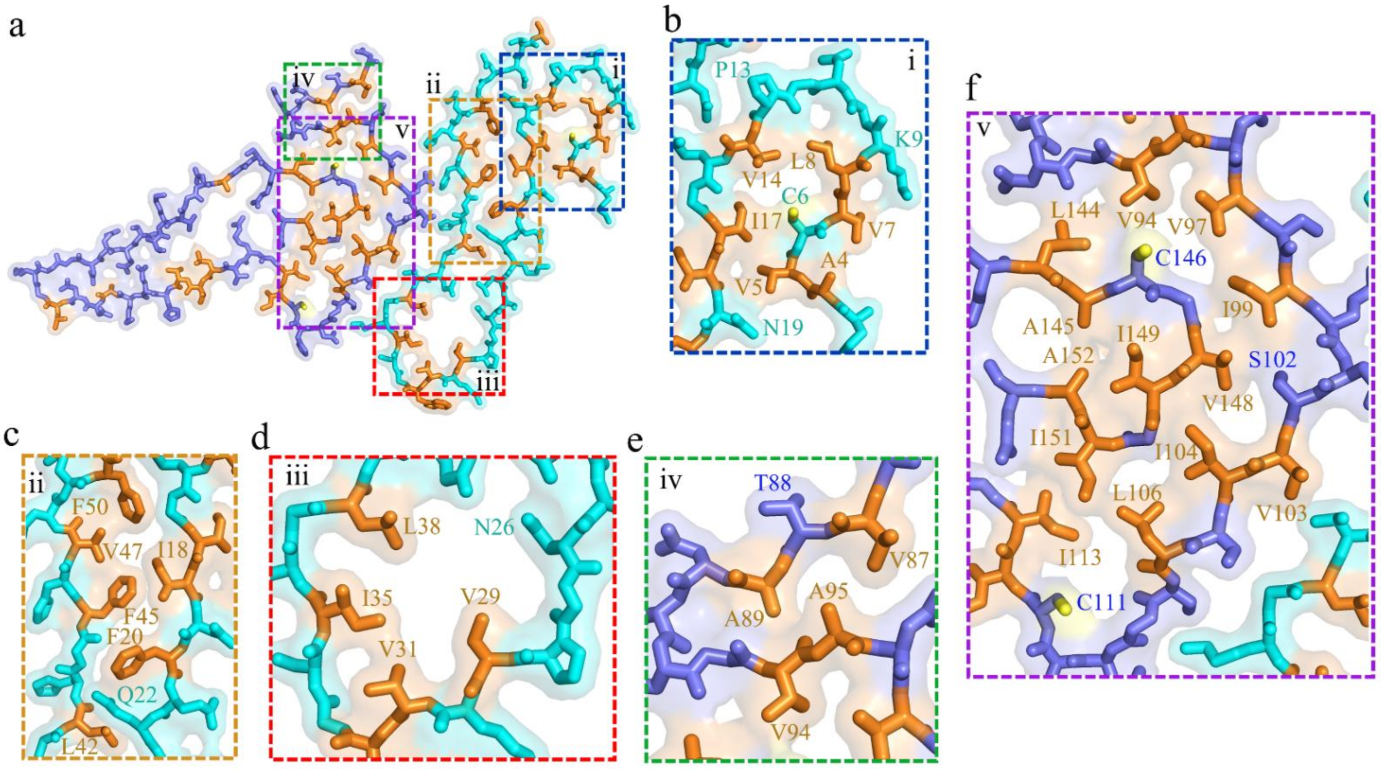

Supplementary Figure 6.

Close-up view of the stick representation of the structure of the SOD1 fibril stabilized by five hydrophobic cavities.

a A space-filled model overlaid onto stick representation of the SOD1 fibril, in which the N-terminal part is shown in cyan and the C-terminal part in blue. Hydrophobic residues and sulfur atoms in Cys6, Cys111, and Cys146 are highlighted in orange and yellow, respectively, and five hydrophobic cavities (i to v) in the SOD1 fibril are magnified in **b** to **f**. **b–f** Magnified top views of the five hydrophobic cavities in the SOD1 fibril, where hydrophobic side chains of Val5, Leu8, Val14, and Ile17, hydrophobic side chains of Ile18, Phe20, Leu42, Phe45, Val47, and Phe50, and hydrophobic side chains of Val29, Leu31, Ile35, and Leu38 are located in the interior of the N-terminal segment of SOD1 fibril to form stable hydrophobic cores, and hydrophobic side chains of Val87, Ala89, and Ala95 and hydrophobic side chains of Val94, Val97, Ile99, Ile104, Leu106, Ile113, Leu144, Ala145, Val148, Ile149, Ile151, and Ala152 are located in the interior of the C-terminal segment to form stable hydrophobic cores.

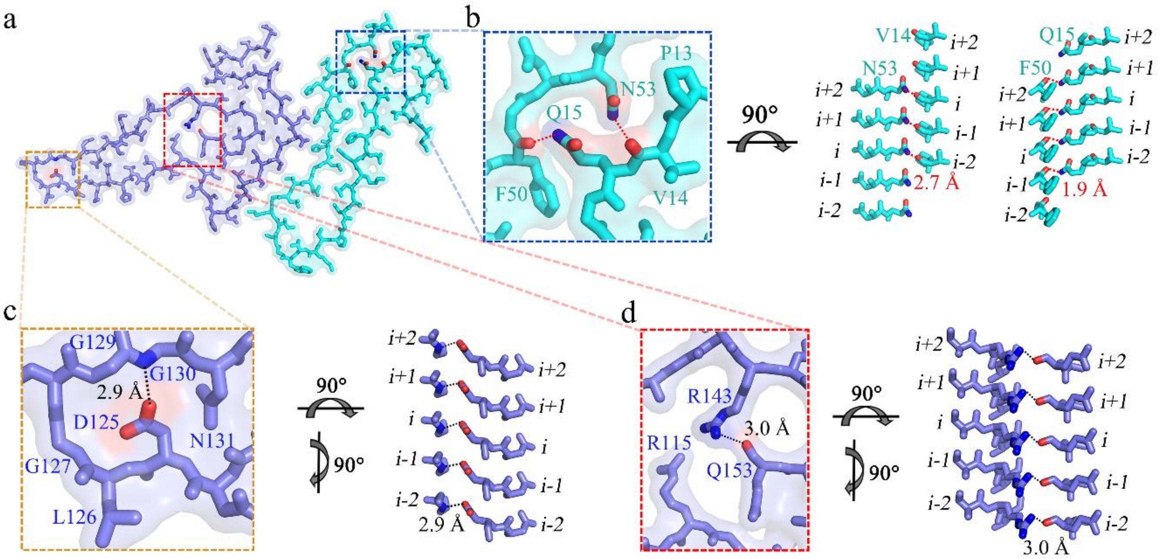

Supplementary Figure 7.

Close-up view of the stick representation of the structure of the SOD1 fibril stabilized by four hydrogen bonds.

a A space-filled model overlaid onto stick representation of the SOD1 fibril, in which the N-terminal segment is shown in cyan and the C-terminal segment in blue. Val/Asn pairs, Phe/Gln pairs, Asp/Gly pairs, and Gln/Arg pairs that form hydrogen bonds are highlighted in red (oxygen atoms in Val, Phe, Asp, and Gln) and blue (nitrogen atoms in Asn, Gln, Gly, and Arg), and three hydrogen bond regions are magnified in **b** to **d**. **b–d** Magnified top views of the three hydrogen bond regions of the SOD1 fibril, where two pairs of amino acids (Val14 and Asn53; and Gln15 and Phe50) from the N-terminal part form two hydrogen bonds, and two pairs of amino acids (Gly130 and Asp125; and Arg143 and Gln153) from the C-terminal part also form two hydrogen bonds. Two side views (right) highlighting a hydrogen bond between Asn53 from the N-terminal part (*i*) and the main chain of Val14 from the molecular layer (*i–2*) of the N-terminal part, with a distance of 2.7 Å (red), or between Gln15 from the N-terminal segment (*i*) and the main chain of Phe50 from the adjacent N-terminal segment (*i–1*), with a distance of 1.9 Å (red). Two side views (right) highlighting a hydrogen bond between Asp125 from the C-terminal part (*i*) and the main chain of Gly130 from the same layer of the C-terminal part (*i*), with a distance of 2.9 Å (black), or between Arg143 from the C-terminal

segment (*i*) and the main chain of Gln153 from the same layer of the C-terminal segment (*i*), with a distance of 3.0 Å (black).

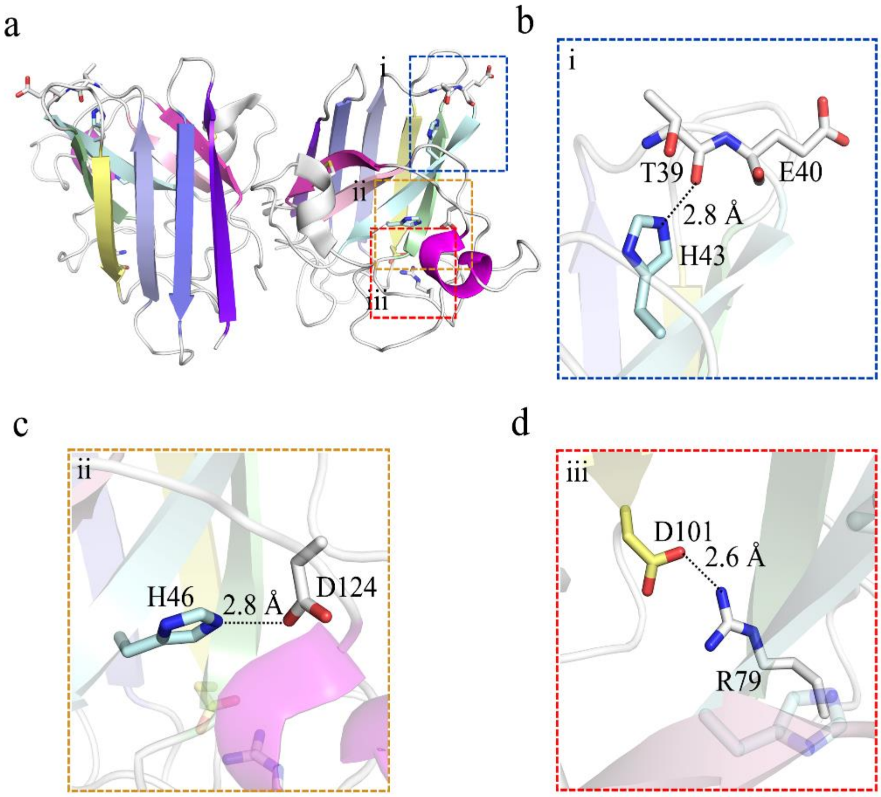

Supplementary Figure 8.

Close-up view of the stick representation of the structure of apo SOD1 dimer.

a Ribbon representation of the structure of full-length apo human SOD1 (1 to 153) dimer with eight β-strands (to form a β-barrel) colored violet, blue, light blue, light cyan, light green, yellow, pink, and light magenta and two α-helices colored gray and magenta in each subunit (PDB 1HL4)[10], and Thr39, Glu40, His43, His46, Arg79, Asp101, and Asp124 in the right monomer are highlighted in stick representation. Thr/His pairs, His/Asp pairs, and Arg/Asp pairs that form hydrogen bonds and salt bridges are highlighted in red (oxygen atoms in Thr, Glu, and Asp) and blue (nitrogen atoms in His and Arg), and three hydrogen bond/salt bridge regions (i to iii) are magnified in **b** to **d**. **b**−**d** Magnified top views of the three regions of the apo human SOD1 monomer, where Thr39 and His43 form a hydrogen bond and two pairs of amino acids (His46 and Asp124; and Arg79 and Asp101) form two salt bridges. Two side views (right) highlighting a hydrogen bond between His43 and the main chain of Thr39, with a distance of 2.8 Å. Two side views (right) highlighting a strong salt bridge

between Asp124 and His46, with a distance of 2.8 Å, or between Asp101 and Arg79, with a distance of 2.6 Å.
