# Supplementary material for: Cryo-EM structure of an amyloid fibril formed by full-length human SOD1 reveals its conformational conversion
Source: Nat Commun. 2022 Jun 17;13:3491. doi: 10.1038/s41467-022-31240-4 (PMC9205981; doi:10.1038/s41467-022-31240-4)
Supplement: Supplementary file 3 — Reporting Summary [file 41467_2022_31240_MOESM3_ESM.pdf]

nature research

Corresponding author(s): Cong Liu & Yi Liang

Last updated by author(s): Jun 6, 2022

Reporting Summary

Nature Research wishes to improve the reproducibility of the work that we publish. This form provides structure for consistency and transparency in reporting. For further information on Nature Research policies, see Authors & Referees and the Editorial Policy Checklist .

Statistics

For all statistical analyses, confirm that the following items are present in the figure legend, table legend, main text, or Methods section.

n/a	Confirmed	
☐	☒	The exact sample size (*n*) for each experimental group/condition, given as a discrete number and unit of measurement
☐	☒	A statement on whether measurements were taken from distinct samples or whether the same sample was measured repeatedly
☐	☒	The statistical test(s) used AND whether they are one- or two-sided<br>*Only common tests should be described solely by name; describe more complex techniques in the Methods section.*
☒	☐	A description of all covariates tested
☒	☐	A description of any assumptions or corrections, such as tests of normality and adjustment for multiple comparisons
☐	☒	A full description of the statistical parameters including central tendency (e.g. means) or other basic estimates (e.g. regression coefficient) AND variation (e.g. standard deviation) or associated estimates of uncertainty (e.g. confidence intervals)
☐	☒	For null hypothesis testing, the test statistic (e.g. *F*, *t*, *r*) with confidence intervals, effect sizes, degrees of freedom and *P* value noted<br>*Give P values as exact values whenever suitable.*
☒	☐	For Bayesian analysis, information on the choice of priors and Markov chain Monte Carlo settings
☒	☐	For hierarchical and complex designs, identification of the appropriate level for tests and full reporting of outcomes
☒	☐	Estimates of effect sizes (e.g. Cohen's *d*, Pearson's *r*), indicating how they were calculated

Our web collection on statistics for biologists contains articles on many of the points above.

Software and code

Policy information about availability of computer code

Data collection	Datasets for cryo-EM were collected with SerialEM 3.8.3.
Data analysis	Cryo-EM data were processed using RELION-3.1, MotionCor2-1.3.2, and CTFFIND4.1.8. Coot 0.8.9.2 was used to build and modify the atomic model of SOD1 fibril. The model was refined using the real-space refinement program in PHENIX 1.15.2. All density map related figures were prepared in Chimera1.15. Ribbon representation of the structure of SOD1 fibril was prepared in PyMol 2.3. AFM images were analyzed by using NanoScope Analysis 2.0 software. The data on cell viability and the TEM images were analyzed by using Origin Pro software version 8.0724.

For manuscripts utilizing custom algorithms or software that are central to the research but not yet described in published literature, software must be made available to editors/reviewers. We strongly encourage code deposition in a community repository (e.g. GitHub). See the Nature Research guidelines for submitting code & software for further information.

Data

Policy information about availability of data

All manuscripts must include a data availability statement. This statement should provide the following information, where applicable:

- Accession codes, unique identifiers, or web links for publicly available datasets
- A list of figures that have associated raw data
- A description of any restrictions on data availability

The cryo-EM density maps of human SOD1 fibril have been deposited in the Electron Microscopy Data Bank (EMDB) under accession code EMD-32227. The coordinates generated in this study are deposited in the Protein Data Bank (PDB) under accession code PDB 7VZF. Previously published structure 1HL4 is available from PDB. The source data underlying Fig. 1a, Supplementary Fig. 1b-f, Supplementary Fig. 2i,j, and Supplementary Fig. 4 are provided as a Source Data.

Field-specific reporting

Please select the one below that is the best fit for your research. If you are not sure, read the appropriate sections before making your selection.

- [x] Life sciences
- [ ] Behavioural & social sciences
- [ ] Ecological, evolutionary & environmental sciences

For a reference copy of the document with all sections, see nature.com/documents/nr-reporting-summary-flat.pdf

Life sciences study design

All studies must disclose on these points even when the disclosure is negative.

Sample size	The amount of cryo-EM micrographs collected was based on the cryo-EM time allocation and previous knowledge estimating that the size is sufficient to generate a high-resolution density. The details of cryo-EM datasets including sample sizes are given in Methods and Supplementary Information. For negative stain EM, the images were collected based on the homogeneous of the fibril estimating that the size is sufficient to get clear views of the SOD1 fibrils. Sample size was chosen based on data variation. Three biologically independent samples were used for TEM imaging (Supplementary Fig. 1a), Congo red binding assays (Supplementary Fig. 1b), mitochondrial dysfunction assays (Supplementary Fig. 2), and AFM imaging (Supplementary Fig. 4) with similar results obtained. Four and six biologically independent samples were used for the MTT assay (Supplementary Fig. 1c,e) and the CCK8 assay (Supplementary Fig. 1d,f), respectively, with similar results obtained.
Data exclusions	No data were excluded from the analyses.
Replication	The Congo red binding experiments were repeated three times with different batches of fibrils and similar results to confirm the reproducibility. The cell viability experiments were repeated four or six times with different batches of fibrils and similar results to confirm the reproducibility. The ultrathin section TEM experiments were repeated three times with different batches of fibrils and similar results to confirm the reproducibility. The AFM and TEM experiments were repeated three times with different batches of fibrils and similar results to confirm the reproducibility. All Biochemical experiments in this paper were repeated independently from 3 to 6 times, and were all successfully reproduced.
Randomization	Our experiments were not related to randomization. Indicated concentrations of proteins were used for biochemical and cellular experiments.
Blinding	Blinding was not applied to our study. For the experiments in our paper, blinding methods were technically not possible: cryo-EM, ultrathin section TEM, Congo red binding assays, and cell viability assays. No animal or human studies were involved.

Reporting for specific materials, systems and methods

We require information from authors about some types of materials, experimental systems and methods used in many studies. Here, indicate whether each material, system or method listed is relevant to your study. If you are not sure if a list item applies to your research, read the appropriate section before selecting a response.

Materials & experimental systems

n/a	Involved in the study	
[x]	[ ]	Antibodies
[ ]	[x]	Eukaryotic cell lines
[x]	[ ]	Palaeontology
[x]	[ ]	Animals and other organisms
[x]	[ ]	Human research participants
[x]	[ ]	Clinical data

Methods

n/a	Involved in the study	
[x]	[ ]	ChIP-seq
[x]	[ ]	Flow cytometry
[x]	[ ]	MRI-based neuroimaging

Eukaryotic cell lines

Policy information about cell lines

Cell line source(s)	SH-SY5Y neuroblastoma cells (catalog number GDC0210) and HEK-293T cells (catalog number GDC0187) were obtained from China Center for Type Culture Collection (CCTCC, Wuhan, China).
Authentication	We verified the SH-SY5Y cell line and and the HEK-293T cell line according to the morphology by light microscopy.
Mycoplasma contamination	SH-SY5Y cell line and HEK-293T cell line were negative in the mycoplasma contamination test.
Commonly misidentified lines (See ICLAC register)	No commonly misidentified lines were used in this study.
